# Predictors of Dietary Supplement Use by U.S. Coast Guard Personnel

**DOI:** 10.1371/journal.pone.0133006

**Published:** 2015-07-31

**Authors:** Krista G. Austin, Lori Lyn Price, Susan M. McGraw, Harris R. Lieberman

**Affiliations:** 1 Military Nutrition Division, US Army Research Institute of Environmental Medicine, Natick, Massachusetts, 01760, United States of America; 2 Oak Ridge Institute for Science and Education, Belcamp, Maryland, 21017, United States of America; 3 Institute for Clinical Research and Health Policy Studies, Tufts Medical Center; Tufts Clinical and Translational Science Institute, Tufts University, Boston, Massachusetts, 02111, United States of America; West Virginia University School of Medicine, UNITED STATES

## Abstract

**Background:**

Personnel in Armed Forces entities such as the US Coast Guard (USCG) engage in strenuous tasks requiring high levels of physiological and psychological fitness. Previous reports have found increased prevalence of dietary supplement (DS) use by military personnel to meet the demands of their occupation.

**Objective:**

This study assessed DS prevalence and patterns of use in USCG personnel and compared these findings to reports from other Armed Forces personnel.

**Design:**

Use of DS by USCG personnel (n = 1059) was assessed by survey at USCG installations. Data were weighted by age, sex, and rank to be representative of total USCG demographics.

**Results:**

Seventy percent of USCG personnel reported using a DS at least 1 time/wk. Thirty-three percent used 1–2 DS ≤ 1 time/wk, 18% 3–4 DS ≥ 1 time/wk, and almost 19% ≥ 5 DS ≥ 1 time/wk. Average expenditure on DSs by UCSG personnel was $40/mo. More than 47% of USCG personnel used a multivitamin and mineral, 33% consumed protein supplements, 22% used individual vitamins and minerals, 23% reported taking combination products, and 9% consumed herbal supplements. Increased use of DS use was associated with high intensity operational occupations, participating in high volumes of aerobic exercise and strength training. Use of DS was not associated with age, education or body mass index.

**Conclusion:**

Occupation is an important determinate of DS use. Prevalence of DS use by USCG personnel is greater than reported for other Armed Forces personnel and reflects high levels of participation in aerobic and strength training activities.

## Introduction

The relationship between occupation and various health-related behaviors has not been an area of extensive investigation. One common self-initiated health-related behavior is consumption of dietary supplements (DS). Dietary supplement use by the United States (U.S.) adult population has steadily risen over the past 20 years [1–2]. National surveys of civilians have shown that 48–53% of adults regularly use DS and overall approximately 64–69% of adults consume DS at least annually [1–4]. Studies of specific occupational subgroups, including military and athletic populations, suggest DS use is more prevalent among individuals who engage in greater amounts of physical activity [5–10] or believe they can gain an advantage in sport or work performance by using DS to modify their body weight and composition [5–10]. Military personnel, like athletes, report greater use of DS products that are marketed to increase fitness and performance such as protein powders and combination products which they believe will help them meet the demands of their occupation [11–13].

The U.S. Coast Guard (USCG) is a military service and is legally defined as an “Armed Force” entity of the United States. Armed Forces personnel regularly engage in physically and mentally demanding tasks [14]. The USCG is responsible for maritime safety and security. The USCG has multiple missions and is also a law enforcement agency. Its personnel can carry firearms, make arrests, and perform law enforcement duties as designated by the Secretary of Homeland Security [15]. In peacetime, the USCG is under the authority of the Department of Homeland Security; in wartime, the USCG is directed by the U.S. Navy [15]. USCG personnel are required to adhere to a variety of regulations like other military services. For example, USCG personnel are required to meet regulations for fitness and body weight standards to remain in service and be eligible for promotion [16,17].

Coast Guardsmen are engaged in a wide-variety of occupational duties. Many of these occupational specialties require substantial physical activity, and USCG personnel may use DS to help meet these demands. For example, they man ships and aircraft and serve as maritime communication and intelligence specialists and experts in weapon systems. Specialty roles include electronic and information system specialists, machinery technicians, damage controlmen and survival technicians. Personnel with administrative and scientific duties provide food and health services, conduct marine science research and provide public affairs services [14,15].

To optimize operational physiological and psychological fitness, the USCG has implemented policies that encourage service members to seek guidance from medical personnel prior to self-administration of DS [18]. Education regarding the effects of DS use and their potential impact on fitness and readiness is also provided to USCG personnel in an attempt to enhance compliance with this policy [18]. Armed Forces personnel, including the USCG, are included in defense health surveillance studies [19], but not in national health surveillance studies, such as the National Health and Nutrition and Examination Survey (NHANES), that monitor the use of DS by the U.S. civilian population [1–4,6]. Given the policies regarding DS implemented by the USCG and the considerable diversity in culture and behavior patterns of each Armed Forces subpopulation, a description of prevalence and patterns of DS use by this population may provide information on various factors that are associated with DS use [19].

This study assessed use of DS, sport nutrition products (drinks, bars and gels) and meal replacement beverages, and reasons for the use of these products by USCG personnel. Furthermore, associations with demographic and lifestyle factors, including sex, age, rank, occupational specialization, marital status, tobacco use, aerobic exercise and participation in strength training were assessed. In addition, we compared use patterns and types of DS consumed by USCG personnel to patterns of DS use by military personnel and the civilian population.

## Methods

### Ethics Statement

This study was approved by the Human Use Review Committee at the U.S. Army Research Institute of Environmental Medicine. No incentives were offered to participants for completion of the survey. Participants completed the survey after an explanation that all information obtained would remain confidential and that participation was voluntary (no identifying data were collected) and they were free to withdraw from the study at any time. Completion and return of the survey served as each participant’s written informed consent. Investigators adhered to U.S. Army Regulation 70–25 and U.S. Army Medical Research and Materiel Command Regulation 70–25 on the use of volunteers in research [20].

### Participants

The final sample obtained for this study consisted of 1,033 Coast Guard personnel. Data were collected from 2010–2011. Sampling was conducted at the following Coast Guard installations: Washington, District of Columbia; New Orleans, Louisiana; Cleveland, Ohio; St Louis, Missouri; San Pedro, California; Seattle, Washington; Alameda, California; Kodiak, Alaska; Honolulu, Hawaii; Miami, Florida; Cape May, New Jersey; Hampton, Virginia; Cape Cod, Massachusetts. Health Promotion Managers approached participants at their respective bases and offered them an opportunity to participate in the study. Users and non-users of DSs were included in the sample.

### Survey

Prior to administering the anonymous survey (Dietary Supplement and Caffeine Intake Survey of U.S. Coast Guard Active Duty Personnel), participants were briefed regarding its contents and appropriate procedures for completing all questions. The survey instrument utilized in the present study was previously developed at the U.S. Army Research Institute of Environmental Medicine for studies related to supplement use by Army Soldiers and was modified in conjunction with USCG staff to reflect population differences. The survey consists of 43 items and included questions on type of DS used, frequency of use (never, 1 time/mo, 1 time/wk, >1 time/wk and daily), reasons for use (general health, enhance performance, improve endurance, increase muscular strength, weight loss, unsure or other) and money spent on DS. Ninety-two individual supplements were listed in the survey including 55 general supplements such as multivitamins, individual vitamins and minerals, combination antioxidants and 37 specific-named products. Selection of branded DS for inclusion was based on then-current patterns of DS purchases at Armed Forces exchange systems and General Nutrition Center stores on or near military installations.

Prior to data analyses, individual supplement and supplement types were grouped into the following categories: multivitamin and multimineral, individual vitamins and minerals, protein/amino acid supplements, combination products, herbal supplements, purported steroid analogs and other based on the definitions provided in Table 1. This is a standardized taxonomy similar to that used in national surveys such as NHANES [12]. Participants were also asked about the use of sports drinks, bars or gels, and meal replacement beverages based on previous reports indicating they are frequently used by military personnel [12]. These nutritional products were categorized separately as they are not regulated as DS as specified by the Dietary Supplement Health and Education Act (DSHEA) of 1994. The survey also collected information on demographic and lifestyle factors including age, sex, body mass index (BMI), education, occupation, marital status, tobacco use and participation in aerobic and strength training exercise. An individual’s occupational specialization was categorized as: 1) operational, which includes jobs such as fireman, damage controlman, maritime law enforcement specialist; 2) support, which includes jobs such as health service technician, food service specialist, electronics technician; and 3) officer, which is a supervisory position.

**Table 1:** Dietary supplement categories as defined in Dietary Supplement and Caffeine Intake Survey of U.S. Coast Guard Active Duty Personnel.

Category	Definition
Dietary Supplement	Any DS^1^ as defined by the DSHEA^2^ legislation
Multivitamin	DS containing two or more vitamins and no additional supplement ingredients
Multimineral	DS containing two or more minerals and no additional supplement ingredients
Protein and Amino Acid	Amino acid mixtures, protein powders, and similar products where the intention is to provide a single or complex protein source
Individual Vitamins or Minerals	DS that were single nutrient ingredient supplements, such as calcium or vitamin D
Combination Products	DS with mixtures of ingredients from any of the above categories; included two or more categories and multiple ingredients
Herbal Supplements	DS that included one or more herbal ingredients with no nutrients or other supplement ingredients; also includes plant-derived ingredients
Purported Steroid Analogs	Steroidal hormones or herbal substitutes for hormones that were marketed as DS and included the Supplement Facts panel on the label

### Data Analyses

Completed surveys were scanned using ScanTools Plus with ScanFlex (version 6.301; Scantron Corporation, Eagan, MN) and data imported into Statistical Package for the Social Sciences (SPSS) (version 20.0; SPSS Inc, Chicago, IL) for conversion to a Statistical Analysis System (SAS) (version 9.2; SAS Institute, Cary, NC) data file for all statistical analyses. Data were weighted prior to analysis to obtain a sample representative of the overall Coast Guard composition as of November 2011 (Table 2). Weights were based on demographic data, including sex, age and rank, obtained from the Defense Manpower Data Center (www.dmdc.osd.mil/) and the characteristics of survey respondents. Survey weightings were calculated by dividing the number of Coast Guard personnel in each cell who were eligible to take the survey by the number in the cell that completed the survey.

**Table 2:** Prevalence (% ± SE) of reported use of any dietary supplement (DS), sports drink, sports bar/gel, and meal replacement beverage among U.S. Coast Guard Active Duty Personnel.

		Estimate of Total Coast Guard Personnel	Any DS	Any Sports Drink	Any Sports Bar Gel etc.	Any Meal Replacement Beverage	Mean Dollar Amount Spent on DS in Past 3 Months	$50 or More Spent on DS Per Month
Characteristic	N	*#*	*% ± SE*	*% ± SE*	*% ± SE*	*% ± SE*	*x ± SE*	*% ± SE*
Total	1033	41,579	69.9±1.50	24.7 ± 1.37	12.0 ± 1.05	9.5 ± 0.96	39.7 ± 2.26	28.7 ± 1.53
**Sex**			** **	** ** **	** **	** **	*	**
Male	855	35,913	69.5±1.65	25.9 ± 1.52	12.3 ± 1.15	9.4 ± 1.05	41.2 ± 2.51	30.0 ± 1.69
Female	178	5,666	72.3±3.35	17.2 ± 2.80	9.9 ± 2.44	9.7 ± 2.24	29.6 ± 4.69	19.9 ± 3.48
**Age (Years)**				**	*		*	**
18 to 24 Years	322	10,200	64.0±2.70	31.5 ± 2.63	11.6 ± 1.78	11.0 ± 1.74	40.9 ± 4.11	32.0 ± 2.80
25 to 29 Years	319	11,240	71.4±2.55	26.2 ± 2.47	10.7 ± 1.75	9.4 ± 1.64	44.1 ± 3.68	34.3 ± 2.85
30 to 39 Years	290	13,861	70.9±2.71	25.1 ± 2.60	15.9 ± 2.19	8.7 ± 1.70	41.9 ± 4.56	28.0 ± 2.79
40+ Years	102	6,278	74.4±4.70	10.3 ± 2.85	6.1 ± 2.38	8.7 ± 3.13	25.5 ± 5.41	15.1 ± 3.83
**Education**								
Some HS/High School	249	9,016	63.5±3.11	23.8 ± 2.78	8.4 ± 1.83	9.1 ± 1.81	41.4 ± 4.57	29.5 ± 3.12
Some College/Associate Degree	566	22,159	71.2±1.94	25.9 ± 1.86	11.8 ± 1.38	8.7 ± 1.18	41.8 ± 3.13	30.8 ± 2.09
Bachelor/Graduate Degree	216	10,335	72.9±3.39	23.2 ± 2.92	15.5 ± 2.55	11.6 ± 2.44	33.8 ± 4.56	23.6 ± 3.17
**Marital Status**				**		**	**	**
Single / Not Married	465	16,713	73.0 ± 2.08	30.0 ± 2.17	13.2 ± 1.62	13.1 ± 1.57	47.2 ± 3.83	35.6 ± 2.44
Married	564	24,723	67.9 ± 2.08	21.2 ± 1.76	10.9 ± 1.36	7.1 ± 1.21	34.5 ± 2.74	23.9 ± 1.94
**Rank**				**			**	**
Enlisted 1–4	437	13,788	66.4±2.27	32.3 ± 2.25	12.9 ± 1.62	11.5 ± 1.52	45.9 ± 3.70	35.6 ± 2.45
Enlisted 5–9	449	19,621	70.8±2.17	22.3 ± 1.99	10.4 ± 1.47	7.9 ± 1.29	41.1 ± 3.63	28.0 ± 2.26
Warrant Officer	26	1,638	76.9±8.23	9.0 ± 6.02	13.5 ± 7.18	0.0 ± 0.00	24.0 ± 8.29	18.3 ± 8.37
Officer	121	6,532	72.8±4.61	20.1 ± 3.69	14.2 ± 3.19	12.4 ± 3.40	26.7 ± 4.37	19.2 ± 3.90
**Occupation** ^1^			**	**			**	**
Operational	430	16,284	74.7±2.15	29.3 ± 2.23	12.5 ± 1.61	10.5 ± 1.48	49.0 ± 3.81	37.1 ± 2.50
Support	298	12,112	62.9±2.85	19.0 ± 2.32	8.7 ± 1.68	7.6 ± 1.56	33.7 ± 4.19	20.3 ± 2.49
Officer	125	6,778	73.5±4.48	19.4 ± 3.56	14.8 ± 3.23	11.9 ± 3.29	28.7 ± 4.50	21.1 ± 4.05
**BMI (kg/m** ^**2**^ **)** ^2^								
18.5 to 24.9	387	14,176	71.2±2.43	26.9 ± 2.33	14.4 ± 1.90	9.2 ± 1.47	34.4 ± 3.49	27.4 ± 2.48
25 to 29.9	549	23,086	69.3±2.05	24.5 ± 1.87	11.2 ± 1.39	9.1 ± 1.29	43.8 ± 3.30	29.9 ± 2.11
30+	93	4,171	68.4±5.12	18.0 ± 3.90	7.5 ± 2.65	10.9 ± 3.70	33.1 ± 5.35	24.1 ± 4.99
**Tobacco Use**								
Currently Use	15	587	59.5 ± 12.96	12.1 ± 8.54	8.2 ± 7.78	4.0 ± 3.96	46.5 ± 18.05	24.5 ± 11.01
Formerly Used	63	2,541	81.7 ± 4.92	24.1 ± 5.51	10.5 ± 4.15	10.3 ± 3.83	28.2 ± 5.38	20.3 ± 5.35
Never Used	954	38,414	69.2 ± 1.57	25.0 ± 1.43	12.2 ± 1.09	9.5 ± 1.00	40.4 ± 2.41	29.2 ± 1.61
**Mean Aerobic Exercise Duration (min)** ^3^ **per week**			**	*	**	**	**	**
0 to 60	74	2,764	38.7±5.81	15.1 ± 4.12	6.7 ± 2.95	2.4 ± 1.70	20.9 ± 8.05	8.3 ± 3.64
61 to 314	263	11,090	71.0±2.87	21.2 ± 2.57	5.5 ± 1.37	6.6 ± 1.69	30.6 ± 4.36	19.4 ± 2.57
315 to 464	212	8,879	69.5±3.38	23.9 ± 3.03	11.6 ± 2.32	10.1 ± 2.38	37.2 ± 4.44	29.7 ± 3.50
465 +	484	18,846	74.0±2.11	28.6 ± 2.10	16.8 ± 1.78	11.9 ± 1.47	48.9 ± 3.47	36.4 ± 2.38
**Participate in Strength Training** ^4^ **each week**			**	**	**	**	**	**
No	208	8,354	46.9±3.64	15.2 ± 2.51	6.9 ± 1.71	4.5 ± 1.37	17.2 ± 2.92	12.6 ± 2.60
Yes	825	33,225	75.7±1.57	27.2 ± 1.59	13.3 ± 1.23	10.7 ± 1.14	44.8 ± 2.66	32.3 ± 1.76

* = P < 0.05

** = P < 0.01.

^1^ Coast Guard occupation is the self-reported area of assignment at the time of the survey: Operational (includes jobs such as fireman, damage controlman, maritime law enforcement specialist); Support (includes jobs such as health service technician, food service specialist, electronics technician); Officer (supervisory positions)

^2^ BMI was calculated from self-reported height and weight.

^3^ Aerobic exercise included percent of individuals who reported nonstop running, cycling, stair climbing, swimming and road marching either within their Coast Guard unit or on their own time each week for the duration ranges: lowest (0–60 minutes), low (61–314 minutes), moderate (315–464 minutes); high (465+ minutes)

^4^ Strength training included percent of individuals who reported lifting weights or other forms of strength condition exercise within their Coast Guard unit or on their own time each week.

Wald chi-square tests were used to test for significant differences in percentages among multiple characteristic levels using the “surveyfreq” procedures (Table 3). Analysis of variance (ANOVA) using the “surveymeans” procedure was used to test mean values across multiple characteristic levels. A p-value of <0.05 was considered statistically significant. Statistical comparisons conducted were pre-planned and restricted to comparisons conducted in a previous study of DS use by other military personnel [12].

**Table 3:** Prevalence (% ± SE) of reported use of number and type dietary supplements (DS) at least once per week or more often over the six months prior to the survey by demographic and lifestyle characteristics among Active Duty U.S. Coast Guard Personnel^1^.

	Number of DS Used Per Week			Dietary Supplements Taken at Least Once a Week						
	1 to 2	3 to 4	5 or more	Multivitamin or Multimineral^3^	Protein & Amino Acid^4^	Individual Vitamins or Minerals^5^	Combination Products^6^	Herbal^7^	Purported Steroid Analogs^8^	Other^9^
Characteristic	*%* ± *SE*	*%* ± *SE*	*%* ± *SE*	*%* ± *SE*	*%* ± *SE*	*%* ± *SE*	*%* ± *SE*	*%* ± *SE*	*%* ± *SE*	*%* ± *SE*
Total (N = 1033)	32.9±1.56	18.4±01.29	18.6±1.26	47.7±1.65	32.7±1.53	22.3±1.36	23.1±1.35	9.4±0.93	1.0±0.32	26.2±1.47
**Sex (%±SE)**				*	** **	**	**			
Male (N = 591)	32.7±1.71	17.3±1.38	19.5±1.39	46.5±1.80	35.1±1.69	20.5±1.44	25.3±1.51	8.9±0.99	1.0±0.35	25.6±1.60
Female (N = 124)	34.1±3.69	25.5±3.43	12.8±2.81	55.0±3.84	17.8±3.02	34.0±3.77	9.1±2.28	12.8±2.61	0.6±0.57	29.8±3.69
**Age**				**	**		**			**
18 to 24 Years (N = 205)	26.0±2.47	17.8±2.18	20.2±2.65	34.9±2.68	40.8±2.78	24.4±2.41	29.0±2.58	12.7±1.86	0.6±0.43	18.1±2.15
25 to 29 Years (N = 228)	34.4±2.68	16.4±2.07	20.7±2.30	50.4±2.82	37.2±2.73	21.3±2.30	26.2±2.50	8.7±1.58	1.6±0.71	26.7±2.50
30 to 39 Years (N = 206)	33.7±2.81	20.3±2.38	17.0±2.24	53.6±2.97	29.0±2.71	20.6±2.39	23.1±2.51	9.3±1.72	1.2±0.67	25.9±2.61
40+ Years (N = 76)	39.8±5.29	19.0±4.31	15.6±3.82	50.4±5.40	19.9±4.42	24.7±4.57	7.7±2.81	5.7±2.47	0.0±0.00	39.0±5.25
**Education**				**						**
Some HS/High School (N = 157)	32.3±3.04	15.7±2.37	15.6±2.36	36.3±3.13	31.5±2.96	20.6±2.66	23.8±2.73	9.3±1.83	0.4±0.42	18.4±2.62
Some College/Associate degree (N = 399)	34.4±2.06	17.6±1.65	19.2±1.71	48.5±2.16	33.3±2.02	23.1±1.84	23.9±1.81	9.8±1.25	1.0±0.41	26.7±1.94
Bachelor/Graduate Degree (N = 158)	30.1±3.56	22.7±3.15	20.0±2.85	55.9±3.78	32.5±3.48	22.3±3.00	20.9±2.94	8.6±2.07	1.4±0.83	32.0±3.51
**Rank**				*	**	*	**	*		
Enlisted 1–4 (N = 289)	28.1±2.17	16.9±1.79	21.4±1.99	40.7±2.37	41.2±2.38	25.2±2.08	28.0±2.18	12.6±1.59	1.0±0.49	21.7±1.99
Enlisted 5–9 (N = 318)	35.9±2.28	18.1±1.83	16.8±1.78	49.6±2.38	29.0±2.15	18.3±1.84	22.8±1.99	8.4±1.33	1.0±0.47	26.7±2.11
Warrant Officer (N = 19)	37.0±9.82	25.2±8.92	14.7±7.01	50.6±10.1	14.5±6.94	30.9±9.32	7.3±5.10	2.8±2.77	0.0±0.00	43.2±10.1
Officer (N = 89)	33.2±4.91	20.9±4.18	18.7±3.83	55.9±5.15	30.7±4.71	26.4±4.40	17.3±3.66	7.5±2.66	1.0±0.98	29.8±4.72
**Occupation**	*	*	*	*	*		**			
Operational (N = 321)	33.6±2.34	19.6±1.95	21.5±2.01	52.1±2.46	37.8±2.37	22.8±2.05	29.1±2.22	10.2±1.48	0.9±0.46	25.6±2.17
Support (N = 186)	31.3±2.75	16.5±2.18	15.1±2.11	44.3±2.93	27.3±2.61	17.6±2.23	18.6±2.30	9.0±1.66	0.4±0.40	24.5±2.55
Officer (N = 348)	33.1±4.82	20.1±4.05	20.2±3.92	57.2±5.04	30.7±4.62	26.5±4.33	17.7±3.65	7.2±2.57	1.0±0.95	30.9±4.69
**BMI** ^**11**^										
18.5 to 24.9 (N = 270)	34.2±2.60	20.1±2.11	16.8±2.03	46.3±2.71	37.7±2.64	22.9±2.24	20.2±2.10	10.2±1.52	1.0±0.53	25.6±2.38
25 to 29.9 (N = 378)	32.6±2.12	18.0±1.80	18.8±1.69	49.1±2.24	30.3±2.00	21.3±1.82	23.3±1.83	8.6±1.20	0.9±0.44	27.2±2.04
30+ (N = 64)	31.3±5.03	15.9±3.70	21.2±4.59	43.2±5.35	28.5±4.95	26.4±4.88	29.7±4.96	11.2±3.86	0.0±0.00	22.3±4.70
**Aerobic Exercise Mean Duration** ^12^ **per Week**	**	**	**	**	**	**	**			**
0 to 60 (N = 29)	20.4±4.82	8.8±3.27	9.5±3.48	21.8±4.96	15.5±4.22	8.18±3.04	13.2±3.97	8.6±3.18	1.4±1.35	15.4±4.34
61 to 314 (N = 181)	41.6±3.31	15.2±2.51	14.2±2.16	45.8±3.32	23.8±2.78	19.4±2.56	15.9±2.25	7.1±1.56	0.4±0.40	22.7±2.78
315 to 464 (N = 147)	32.8 ±3.41	17.2±2.66	19.5±3.06	50.1±3.67	32.2±3.45	26.5±3.30	23.3±3.15	9.2±2.23	1.1±0.81	22.5±3.30
465+ (N = 358)	29.7±2.16	22.3±2.00	22.0±1.92	51.4±2.37	40.8±2.31	24.1±2.00	28.6±2.09	11.0±1.42	1.2±0.48	31.5±2.22
**Participate in Strength Training** ^13^ **each Week**		**	**	**	**	**	**	**		*
No (N = 96)	30.5 ± 3.40	8.1±1.92	8.3±1.97	30.4±3.31	9.8±2.28	15.5±2.57	4.6±1.37	5.2±1.49	0.9±0.62	19.7±2.93
Yes (N = 619)	33.5 ±1.75	21.0±1.52	21.1±1.48	52.0±1.84	38.5±1.77	24.0±1.57	27.7±1.61	10.5±1.10	1.0±0.36	27.8±1.68

* = p < 0.05

** = p < 0.01

Analyses are presented as frequency (or mean) ± standard error. Standard errors were estimated using a Taylor series linearization method that incorporated sampling weights. Logistic regression models using the “surveylogistic” procedure examined relationships between measures of DS use and demographic characteristics of Coast Guard personnel including age, sex, education, marital status, BMI, tobacco use, as well as aerobic exercise duration and strength training (Table 4). Models were adjusted for sex, age and rank. Categorical levels of rank and education variables were highly related, therefore, analyses were performed on models adjusted only for age, sex, and rank. Results of multivariate logistic regression are presented as odds ratios (OR) and 95% confidence intervals (CIs). The use of the survey procedures allowed for sample weights to be applied to all analyses.

**Table 4:** Association of number and type of dietary supplement (DS) use at least once per week over the previous six months by supplement ingredients and amount spent per month on DS with selected demographic and lifestyle characteristics.

	Dietary Supplements Taken at Least Once a Week						
	Any DS	5 or More DS	Multivitamin or Multimineral	Protein & Amino Acid	Combination Products	Herbal	$50 or more Spent on DS/month
Characteristic							
**Sex**		*					
Male	1.0	1.0	1.0	1.0	1.0	1.0	1.0
Female	1.21 (0.84, 1.73)	0.56* (0.33, 0.98)	1.53* (1.09, 2.14)	0.34** (0.21, 0.53)	0.25** (0.14, 0.45)	1.40 (0.82, 2.41)	0.54* (0.33, 0.87)
**Age (Years)**							
18 to 24 Years	0.65 (0.34, 1.25)	1.20 (0.56, 2.60)	0.53* (0.29, 0.95)	2.33* (1.17, 4.65)	4.93** (2.00, 12.15)	1.37 (0.44, 4.33)	2.00 (0.96, 4.16)
25 to 29 Years	0.91 (0.51, 1.64)	1.36 (0.67, 2.76)	1.01 (0.60, 1.71)	2.21* (1.18, 4.15)	4.16** (1.79, 9.66)	1.08 (0.37, 3.20)	2.52** (1.29, 4.91)
30 to 39 Years	0.88 (0.49, 1.56)	1.13 (0.57, 2.26)	1.16 (0.70, 1.92)	1.61 (0.87, 3.00)	3.44** (1.50, 7.92)	1.41 (0.49, 4.03)	2.08* (1.09, 3.98)
40+ Years	1.0	1.0	1.0	1.0	1.0	1.0	1.0
**Education**							
Some HS/High School	1.0	1.0	1.0	1.0	1.0	1.0	1.0
Some College/ Associate Degree	1.32 (0.95, 1.84)	1.43 (0.93, 2.20)	1.44* (1.04, 2.00)	1.34 (0.96, 1.87)	1.20 (0.82, 1.75)	1.20 (0.70, 2.06)	1.18 (0.82, 1.70)
Bachelor/ Graduate Degree	1.30 (0.77, 2.19)	1.85 (0.93, 3.69)	1.67* (1.02, 2.73)	1.94* (1.13, 3.36)	1.87* (1.05, 3.33)	1.35 (0.59, 3.05)	1.28 (0.74, 2.21)
**Marital Status**							
Single/Not Married/ Widowed/Divorced	1.0	1.0	1.0	1.0	1.0	1.0	1.0
Married	0.63** (0.46, 0.85)	0.58** (0.40, 0.84)	0.62** (0.46, 0.84)	0.65** (0.47, 0.90)	0.56**(0.39, 0.80)	0.90 (0.56, 1.44)	0.57** (0.40, 0.80)
**BMI (kg/m** ^**2**^ **)**							
18.5 to 24.9	1.0	1.0	1.0	1.0	1.0	1.0	1.0
25 to 29.9	0.85 (0.62, 1.17)	1.21 (0.82, 1.79)	1.03 (0.76, 1.39)	0.75 (0.54, 1.04)	1.30 (0.92, 1.85)	0.98 (0.62, 1.53)	1.20 (0.86, 1.68)
30+	0.79 (0.45, 1.36)	1.51 (0.79, 2.88)	0.77 (0.46, 1.27)	0.75 (0.42, 1.31)	1.99* (1.10, 3.60)	1.42 (0.60, 3.32)	0.95 (0.51, 1.79)
**Tobacco Use**							
Don’t Currently Use	1.0	1.0	1.0	1.0	1.0	1.0	1.0
Currently Use	0.64 (0.22, 1.83)	1.06 (0.29, 3.86)	1.20 (0.42, 3.42)	0.75 (0.22, 2.56)	0.77 (0.20, 2.88)	2.13 (0.57, 8.01)	0.85 (0.25, 2.92)
**Aerobic Exercise Duration** ^**1**^							
Low exercise	1.0	1.0	1.0	1.0	1.0	1.0	1.0
Medium exercise	1.38 (0.95, 1.99)	1.19 (0.78, 1.82)	1.27 (0.90, 1.78)	1.57* (1.09, 2.24)	1.45 (0.98, 2.15)	0.96 (0.54, 1.70)	1.50* (1.02, 2.20)
High exercise	2.04** (1.25, 3.33)	2.08** (1.35, 3.21)	2.00** (1.30, 3.06)	2.10** (1.42, 3.11)	1.88** (1.25, 2.81)	1.43 (0.80, 2.53)	2.11** (1.41, 3.14)
**Strength Training**							
No	1.0	1.0	1.0	1.0	1.0	1.0	1.0
Yes	3.82** (2.72, 5.38)	2.81** (1.64, 4.81)	2.65** (1.87, 3.77)	5.47** (3.15, 9.48)	7.64** (3.98, 14.68)	2.14* (1.13, 4.04)	3.09** (1.88, 5.08)

* = p< 0.05

** = p < 0.01.

## Results

### Population Demographics

Participants in the present study were 86% male and 14% female. The average (mean ± SD) age was 30.6 ± 8.0 years and the average BMI was 26.1 ± 3.0. Sixty percent of respondents were married. Twenty-two percent of participants reported having some high school education, 53% some college or an associate’s degree and 25% a bachelor or graduate degree. Eighty percent of participants reported performing strength training at least one time per week and the average amount of time spent performing aerobic exercise was 273 ± 230.3 minutes. The demographic characteristics of DS users can be found in Table 2.

### Prevalence and Patterns of DS Use

Seventy percent of USCG personnel reported using a DS at least one time per week (≥ 1 time/wk) in the 6 months prior to the survey. Of those respondents, 33% reported using 1–2 DS ≥ 1 time/wk, 18% reported the use of 3–4 DS ≥ 1 time/wk, and 19% reported taking ≥ 5 DS ≥ 1 time/wk (Table 3). Forty-eight percent of USCG personnel reporting the use of DS chose to use a multivitamin and mineral ≥ 1 time/wk, 33% consumed a protein and amino acid DS, 22% used an individual vitamin or mineral, 23% reported taking combination products ≥ 1 time/wk, 9% consumed a herbal, 1% reported the use of purported steroid analogs ≥ 1 time/wk and 26% of respondents reported the use of DS classified as ‘other’ (Table 3). The average expenditure on DS in the 3 months prior to the survey was $40 and more than 25% of personnel spent >$50/mo on DS.

Comparison of DS prevalence and patterns by demographic and lifestyle characteristics identified a number of consistent predictors of DS use. Prevalence of any DS use, number of DS used per week, financial expenditure on DS, multivitamin and mineral, protein and amino acid, individual vitamins or minerals, combination products and supplements classified as ‘other’ was higher among respondents who were performing more than 60 minutes of aerobic exercise training per week (p<0.01) or strength training (p<0.05). Use of any DS was not significantly associated with the characteristics of education, BMI, marital status or the use of tobacco products, but was significantly higher among USCG personnel who worked as an officer or in an operational occupation when compared to support personnel (p<0.01). The amount of money spent on DS was significantly greater in personnel who were male (p<0.05), younger (p<0.05), single (p<0.01), or whose occupation was operational in nature (p<0.01).

Further analyses of individual DS classes identified specific demographic and lifestyle predictors associated with patterns of DS use (Table 3). Multivitamin and mineral use was significantly greater in participants above the age of 24 (p<0.01) and use prevalence increased with level of education obtained by USCG personnel (p<0.01). The use of individual vitamins or minerals was significantly greater in males (p<0.01). Prevalence of protein and amino acid use was higher in males (p<0.01), greatest among those personnel ages 18–24y (p<0.01) and participants who were lower rank enlisted personnel (Enlisted pay grade1 (E1)-Enlisted pay grade 4 (E4); p<0.01). Use of combination products was significantly greater in males (p<0.01) and among operational personnel such as law enforcement and operations specialist (p<0.01), but was significantly less among those who were 40 years or older (p<0.01). Herbals were more likely to be used by participants with ranks of E1-E4 (p<0.05) or by those who regularly performed strength training (p<0.01). Supplement use categorized as ‘other’ was more prevalent among individuals who had a more advanced educational attainment (p<0.01) and were less likely to be used by participants aged 18–24 (p<0.01).

### Prevalence and Predictors of Sport Nutrition Product Use

Approximately 25% of respondents consumed sports drinks ≥ 1 time/wk, 12% consumed sports bars or gels and almost 10% consumed a meal replacement beverage (MRB) (Table 2). Use of any sport nutrition product was consistently associated with performing more than 60 minutes of aerobic exercise per week (p<0.05) or participation in strength training (p<0.01). The different forms of sport nutrition products were associated with specific demographic predictors of use. Sport drink use was more prevalent among males (p<0.01), younger personnel (p<0.01), participants who were single (p<0.01) or operational personnel (p<0.01). Participants greater than 40 years of age were less likely to use sports drinks, bars or gels (p<0.05) and users of meal replacement beverages were more likely to be single (p<0.05).

### Multivariate Analyses of DS Use

All models were adjusted for the demographic characteristics of sex, age and rank logistic regression (Table 4). Female personnel remained less likely than males to use 5 or more DS per week (p<0.05), protein and amino acid DS (p<0.01) or combination products, but were one and a half times more likely to use a multivitamin and mineral DS (p<0.05). Respondents between the ages of 18–24 and 25–29 years of age were more than twice as likely to use protein and amino acid DS and combination products compared to those >40 years. Education continued to be a significant predictor of multivitamin and mineral and protein and amino acid DS use. Participants with at least some college (p<0.05) or a bachelor or graduate degree (p<0.05) were more likely to use a multivitamin and mineral compared to those with a high school education. Those with an advanced degree were also more likely to be users of protein and amino acid DS (p<0.05) compared to those with a high school education or less. Married personnel were less likely than single participants to use any DS (p<0.01), including a multivitamin and mineral (p<0.01), protein and amino acid DS (p<0.01) or combination product (p<0.01) and to take 5 or more supplements weekly (p<0.01). Respondents who engaged in high volumes of aerobic exercise or strength training were twice as likely to use a DS (p<0.01), including multivitamins and minerals (p<0.01), protein and amino acid DS (p<0.01) and combination products. They were also more likely to consume 5 or more supplements weekly (p<0.01), compared to those reporting low volumes of aerobic exercise or not participating in strength training. Participation in strength training was also significantly associated with a greater use of herbal DS (p<0.05). Regression modeling indicated that predictors of high DS expenditures (>$50/mo) included being male (p<0.05), single (p<0.01), 25–39 years old (compared to >40) (p<0.05), engaging in moderate (p<0.05) to high (p<0.01) volumes of aerobic exercise or participating in strength training (p<0.01).

### Reasons for DS Use

Reasons for the use of DS are reported in Table 5. Among supplement users, the most common reason cited for DS use was to promote general health (55%), followed by increasing muscle strength (24%), providing more energy (20%), enhancing performance (18%) or ‘other’ (16%). Users of multivitamins and minerals, individual vitamins and minerals, herbals and ‘other’ primarily reported using these DS classes to promote general health. However, users of protein and amino acid DS primarily used this class of DS to increase muscular strength (21%). Participants reporting the use of combination products stated they did so to enhance performance (10%).

**Table 5:** Reported reasons for using any dietary supplement (DS) and specific dietary supplement types at least once per week over the six months prior to the survey (prevalence % ± SE).

	Any DS	Multivitamin or Multimineral	Protein & Amino Acids	Individual Vitamins or Minerals	Combination Supplements	Herbals	Purported Steroid Analogs	Other
Reported Reasons for DS Use	*% ± SE*	*% ± SE*	*% ± SE*	*% ± SE*	*% ± SE*	*% ± SE*	*% ± SE*	*% ± SE*
Promote General Health	55.6 ± 1.63	42.9 ± 1.64	7.6 ± 0.82	17.7 ± 1.25	2.9 ± 0.50	4.9 ± 0.72	0.2 ± 0.14	17.6 ± 1.32
Give More Energy	20.7 ± 1.29	3.0 ± 0.53	3.2 ± 0.54	3.1 ± 0.57	7.4 ± 0.81	1.8 ± 0.39	0.0 ± 0.00	5.2 ± 0.69
Greater Muscle Strength	24.1 ± 1.37	1.6 ± 0.37	21.6 ± 1.31	0.3 ± 0.16	8.5 ± 0.87	0.7 ± 0.25	0.1 ± 0.12	0.6 ± 0.24
Performance Enhancer	18.5 ± 1.25	1.9 ± 0.41	8.5 ± 0.88	0.8 ± 0.25	10.1 ± 0.97	1.2 ± 0.32	0.2 ± 0.12	2.0 ± 0.41
Weight Loss	9.4 ± 0.98	1.1 ± 0.31	2.3 ± 0.52	0.4 ± 0.19	4.2 ± 0.63	0.2 ± 0.12	0.0 ± 0.00	0.8 ± 0.28
Increased Endurance	11.6 ± 1.04	1.2 ± 0.33	4.6 ± 0.68	0.9 ± 0.27	4.7 ± 0.65	0.4 ± 0.18	0.0 ± 0.00	0.9 ± 0.30
Not Sure	7.0 ± 0.81	1.1 ± 0.31	0.1 ± 0.08	0.8 ± 0.26	0.8 ± 0.28	0.7 ± 0.24	0.0 ± 0.00	0.7 ± 0.25
Other	16.3 ± 1.20	0.8 ± 0.27	2.6 ± 0.52	2.1 ± 0.43	1.2 ± 0.35	2.8 ± 0.52	0.1 ± 0.08	1.8 ± 0.42

## Discussion

The present study is the first to report prevalence of DS use of the USCG. Self-reported use of DS was greater among USCG personnel (70%) than has previously been cited for Army, Air Force, Navy and Marine (55%-60%) personnel [21]. Regular use by USCG was higher than reported for adult civilians (54%); however, annual use (64–69%) was similar [1–4]. Prevalence of using 1–2 DS per week was 32% among USCG personnel which is similar to earlier reports of active duty Soldiers and civilians [2–4, 12]. However, 18% of respondents used of 5 or more DS per week which is greater than previously reported for active duty Soldiers (12%) [12]. Patterns of DS use among USCG were comparable to previous reports of active duty Soldiers with multivitamins and minerals and protein and amino acid supplement use being the most common forms of DS consumed; however, overall use prevalence by USCG personnel was higher than Soldiers, especially of protein and amino acid DS. Dietary supplement use was associated with occupational specialization requiring increased levels of energy expenditure and participation in aerobic exercise and strength training but was not associated with age, education, BMI or tobacco use as previously reported for other military and civilian populations [6,12]. This study indicates that individuals engaged in an occupation requiring physical fitness and regular assessment of physical fitness will consume greater amounts of DS than the general population. Other populations, such as police, firefighters, construction and mining trades may also have unique patterns of DS consumption and their use of DS should be investigated.

The greater use of DS by USCG personnel when compared to active duty Soldiers is attributable to a higher use prevalence of multivitamin and minerals, protein and amino acids, individual vitamins and minerals and combination products. The prevalence of purported steroid analogs and herbal use was similar when USCG personnel are compared to Army personnel [12]. Longitudinal data from the general civilian population has identified an increased use of specific DS classes over time, including protein and amino acid supplements, fish oils and herbals [2,6], and recent studies of recreational athletes and regular gym users also report an increased use of protein and amino acids supplements and combination products [22, 23]. Use of protein supplements is much higher in both the USCG (33%) and Army population (19%) than the general civilian population (1%) [2,12]. Differences between USCG and Army personnel in use of certain categories of DS may be a function of their overall increasing popularity within the American culture and the influence of our respondent’s work environment (e.g. high levels of physical activity, body weight and composition requirements). Reported use of herbals by USCG personnel was similar to the civilian population (9% vs. 8%); however reasons for herbal use were not similar between populations [2]. Approximately 27% of civilians report the use of herbals to improve health [2], whereas only 5% of USCG respondents reported use of herbal DS for health purposes. In addition, herbal supplement use is associated in the civilian population with being uninsured, using more medications and specific health-related concerns (e.g. heart health or lowering cholesterol) [2]. Thus, use of herbal DS for health purposes may be less prevalent within the USCG population as a result of overall better health, participating in more physical labor, being insured and being a younger, healthier population given the age, weight and fitness requirements necessary for active duty.

In addition to assessing DS use, we also assessed use of sport nutrition products by USCG personnel. Almost twenty-five percent of respondents reported the use of sports drinks which is similar to the findings of Lieberman et al. [12] for the Soldier population (23%). Use of sports bars or gels and meal replacement beverages was substantially higher by USCG respondents, 12% vs. 6% and 10% vs. 3%, respectively. Similar to Soldiers [6], use of these products increased when greater amounts of aerobic exercise were performed each week and use was also associated with participation in strength training. Therefore, greater use of sports nutrition products by USCG personnel is associated with an increased number participating in high levels of physical activity. This relationship is also relevant to DS use, as the increased prevalence of multivitamin and mineral, protein and amino acid and combination DS use was higher in those reporting greater participation in aerobic and strength training exercise; thus, increased use may be a direct function of the active lifestyles lead by USCG personnel. Our findings are further supported by the civilian literature which reports a greater use of specific DS classes and sport nutrition products among more health-oriented and physically active individuals [4, 22–23].

The most common reason cited for overall DS use by the USCG population was to improve general health. This finding is consistent with reasons for DS use cited by civilian and military populations [2, 12]. Multivitamins and minerals and individual vitamin and mineral DS are the two primary DS classes that USCG, military and civilian populations report they use to improve overall health [2, 12]. Similar to other studies of uniformed personnel, protein supplements and combination products were reported to be used by USCG personnel to promote gains in muscle strength, increase endurance, provide more energy and facilitate weight loss. Reasons for the use of protein supplements and combination products by USCG personnel were similar to those reported by athletes [24] but differed from civilians who cite the primary reason for use of PS as improving overall health [2]. Civilians do not generally report the use of DS for reasons related to physical performance or weight modification [2]. Such differences between the general civilian population and USCG personnel are most likely a function of physical activity levels and occupational requirements, such as the mandatory weight and fitness standards of the USCG.

Unlike other studies of uniformed personnel, we observed greater use of DS by certain occupational subgroups, specifically heavy use of protein and amino acid supplements and combination products by operational personnel. Operational personnel of the USCG are responsible for performing high tempo operations on a daily basis such as security boarding of suspicious vessels, search and rescue missions, military tactical operations, and drug interdiction. These tasks can result in a high level of energy expenditure and thus may promote increased use of DS by occupations which believe DS sustain physical and mental energy levels, an observation that is consistent with the reasons USCG personnel cite for use of protein and amino acid DS and combination products.

On average, USCG personnel spent $40 every 3 months on DS, and almost 29% spent $50 or more per month which is similar to expenditures reported by active duty Soldiers (12). Females, older respondents, married personnel and officers in the USCG were less likely to spend $50 or more per month. Similar to the Army population, sex, age, rank, participation in aerobic exercise of long durations and strength training influenced the level of financial expenditure by USCG personnel on DS and the number of supplements taken each week.

Education efforts by the USCG include course instruction on the use of dietary supplements and the unknown health effects of some DS classes [16]; however, since many USCG service members use substantial quantities of DS, especially combination products, further efforts to educate personnel are warranted. While scientific evidence for the use of vitamin and mineral DS to address nutritional deficiencies is readily available [25, 26], there is limited evidence regarding the safety and efficacy of many ingredients contained in combination product and herbal DS [27–28]. As such, increased awareness of ingredients in DS that may be harmful is necessary to protect the health and well-being of service members. Educational tools, such as the Operation Supplement Safety website, provided by the DoD Human Performance Resource Center [29], could provide USCG personnel guidance regarding potentially harmful DS. Since many service members wish to improve their performance and obtain health benefits associated with increased physical fitness, further efforts are necessary to provide personnel with information on how to obtain such benefits using performance training and nutrition.

## Limitations & Strengths

While the present descriptive analyses provide insight into patterns and prevalence of DS use by a unique arm of the Armed Forces, limitations of this cross-sectional study design must be acknowledged. First, the prevalence and patterns of DS use by this group was determined in a convenience sample that was not random or stratified potentially leading to bias because participants willing to complete surveys or have a strong belief regarding DS use or health could be more likely to participate. In addition, as with all surveys involving self-report, the present study may also reflect recall bias since the participants were requested to recall DS used over the prior 6 months. Despite these limitations, the present study has multiple strengths including the use of a large sample, application of survey weights, and use of multivariate analyses to arrive at conclusions.

## Conclusion

In summary, seventy percent of USCG personnel reported regular use of DS which is greater than previously reported for military services and US civilians. The greater prevalence of DS use is attributable to increased use of multivitamins and minerals, protein and amino acid supplements, individual vitamins and minerals and combination products, and is associated with participation in high volumes of aerobic exercise and strength training. These findings highlight the need for randomized clinical trials to evaluate the efficacy and safety of many currently available DS on the market. Given limited FDA regulation of DS and the unknown health effects of consuming ergogenic and botanical ingredients contained in some DS, USCG personnel should be advised to seek guidance from healthcare personnel before initiating DS use. All Armed Forces personnel would benefit from further guidance on the risks and benefits of DS use.
